# A Comparative Study of Noninvasive Hypoxia Imaging with ^18^F-Fluoroerythronitroimidazole and ^18^F-Fluoromisonidazole PET/CT in Patients with Lung Cancer

**DOI:** 10.1371/journal.pone.0157606

**Published:** 2016-06-20

**Authors:** Yuchun Wei, Wei Zhao, Yong Huang, Qingxi Yu, Shouhui Zhu, Suzhen Wang, Shuqiang Zhao, Xudong Hu, Jinming Yu, Shuanghu Yuan

**Affiliations:** 1 School of Medicine and Life Sciences, University of Jinan-Shandong Academy of Medical Sciences, Jinan, China; 2 Department of Radiation Oncology, Shandong Cancer Hospital, Shandong University, Jinan, China; 3 Department of Nuclear Medicine, Shandong Cancer Hospital, Shandong University, Jinan, China; Stanford University, UNITED STATES

## Abstract

**Purpose:**

This is a clinical study to compare noninvasive hypoxia imaging using ^18^F-fluoroerythronitroimidazole (^18^F-FETNIM) and ^18^F-fluoromisonidazole (^18^F-FMISO) positron emission tomography/computed tomography (PET/CT) in patients with inoperable stages III–IV lung cancer.

**Methods:**

A total of forty-two patients with inoperable stages III–IV lung cancer underwent ^18^F-FETNIM PET/CT (n = 18) and ^18^F-FMISO PET/CT (n = 24) before chemo/radiation therapy. The standard uptake values (SUVs) of malignant and normal tissues depict ^18^F-FETNIM PET/CT and ^18^F-FMISO PET/CT uptake. Tumor-to-blood ratios (T/B) were used to quantify hypoxia.

**Results:**

All patients with lung cancer underwent ^18^F-FETNIM PET/CT and ^18^F-FMISO PET/CT successfully. Compared to ^18^F-FMISO, ^18^F-FETNIM showed similar uptake in muscle, thyroid, spleen, pancreas, heart, lung and different uptake in blood, liver, and kidney. Significantly higher SUV and T/B ratio with ^18^F-FMISO (2.56±0.77, 1.98±0.54), as compared to ^18^F-FETNIM (2.12±0.56, 1.42±0.33) were seen in tumor, *P =* 0.022, <0.001. For the patients with different histopathological subtypes, no significant difference of SUV (or T/B ratio) was observed both in ^18^F-FMISO and ^18^F-FETNIM in tumor. A significantly different SUV (or T/B ratio) was detected between < = 2cm, 2~5cm, and >5cm groups in ^18^F-FMISO PET/CT, *P =* 0.015 (or *P* = 0.029), whereas no difference was detected in ^18^F-FMISO PET/CT, *P =* 0.446 (or *P* = 0.707). Both ^18^F-FETNIM and ^18^F-FMISO showed significantly higher SUVs (or T/B ratios) in stage IV than stage III, *P* = 0.021, 0.013 (or *P* = 0.032, 0.02).

**Conclusion:**

^18^F-FMISO showed significantly higher uptake than ^18^F-FETNIM in tumor/non-tumor ratio and might be a better hypoxia tracer in lung cancer.

## Introduction

Intratumoral hypoxia increases tumor aggressiveness, chemo- and radio-resistance. Locoregional failure is common in several human cancers, particularly after chemoradiotherapy, and may be attributed to intrinsic tumor resistance to radiotherapy and/or chemotherapy. Hypoxia status has a prognostic value and therapeutic implications [1]. Consequently, multiple methods have been proposed to measure hypoxia, both invasively and noninvasively. Although the invasive polarographic needle electrode method is regarded as the gold standard, this method is invasive, difficult for deep tumor tissues, and represents only a small section of the tumor, often misrepresenting the severity of the tumor hypoxia and resulting in an incorrect prognosis [2]. Recently, PET radiotracers have been intensively investigated as the noninvasive methods for imaging hypoxia within the tumor microenvironment, the common radiotracers including ^18^F-fluoroerythronitroimidazole (^18^F-FETNIM) and ^18^F-fluoromisonidazole (^18^F-FMISO).

^18^F-FMISO PET has been widely used to detect tumor hypoxia in clinical [3,4]. Given the pivotal role of tumor hypoxia in cancer treatment response and prognosis, ^18^F-FMISO PET seems to be a very promising modality in the evaluation of oncological patients. Nevertheless, despite its wide application, ^18^F-FMISO remains a tracer, whose role in the diagnostic workup of patients with lung cancer is still open. On the other hand, ^18^F-FETNIM, a radiotracer able to detect hypoxia, has mainly been studied in head and neck cancers, and it seems that high uptake of 18F-FETNIM is associated with a poor outcome in head-and-neck tumors [5]. Recently, in the study of non-small cell lung cancer, ^18^F-FETNIM tumor/blood ratio (T/Bmax) and hypoxia volume (HV) were strong predictors for overall survival (OS) after treatment, and ^18^F-FDG uptake of the primary lesions did not have a significant relationship with survival [6]. These original observations prompted us to study the uptake and metabolism of ^18^F-FMISO and ^18^F-FETNIM in tumor-bearing mammals [7] and, finally, in cancer patients.

In this pilot study, we compared the noninvasive hypoxia vivo imaging ability of ^18^F-FMISO PET/CT with that of ^18^F-FETNIM PET/CT in patients with lung cancer and aimed to find the better noninvasive method for evaluation of hypoxia in lung cancer. A further aim of the study was to assess the biodistribution of the two radiotracers in normal tissues.

## Materials and Methods

### Ethical approval

The study was approved by the Ethical Committee of the Shandong Cancer Hospital and Institute, and each patient gave written and informed consent before the study.

### Patients

Forty-two patients inoperable stage III–IV lung cancer were enrolled in this prospective study before chemo/radiation therapy between February 2014 and August 2015 in Shandong Cancer Hospital. All patients received conventional physical examinations, laboratory tests, and systematically diagnostic staging procedures including enhanced CT, magnetic resonance imaging (MRI), and single photon emission computed tomography (SPECT) bone scan and ultrasonography. The clinical stage was determined by 2010 American Joint Committee on Cancer TNM classification system. The final staging was based on the findings at pathologic examination. They were randomized into two groups, one group of twenty-four patients (twenty-one male, three female) were imaged with ^18^F-FMISO PET/CT, and another group of eighteen patients (fourteen male, four female) with ^18^F-FETNIM PET/CT imaging. The study was approved by the Ethical Committee of the Shandong Cancer Hospital and Institute, and each patient gave written and informed consent before the study.

### ^18^F-FMISO and ^18^F-FETNIM PET/CT imaging

^18^F-FMISO and ^18^F-FETNIM PET/CT studies were performed before any treatment. All patients underwent PET/CT scan 1 to 7 days before treatment. ^18^F-FMISO and ^18^F-FETNIM was prepared as described in previous studies [7,8]. The radiochemical purity of ^18^F-FMISO and ^18^F-FETNIM exceeded 95%, and its specific radioactivity exceeded 37 GBq (1,000 mCi)/μmol. No specific subject preparations, patients did not need fasting and did not receive CT contrast agents. Patients received a dose of 4.81-MBq/kg (0.13 mCi/kg) ^18^F-FMISO and ^18^F-FETNIM intravenously and then rested for approximately 120 minutes [9]. Scanning of chest was performed with an integrated PET/CT device (Discovery LS; GE Healthcare). The spiral CT component was performed with an x-ray tube voltage peak of 140 kV and 80 mA, a 6:1 pitch, a slice thickness of 4.25 mm, and a rotation speed of 0.8 s per rotation. A full-ring dedicated PET scan of the same axial range followed. The patients were in normal shallow respiration during image acquisition. The images were attenuation corrected with the transmission data from CT. The attenuation-corrected PET images, CT images, and fused PET/CT images displayed as coronal, sagittal, and transaxial slices were viewed on a Xeleris workstation (GEHealthcare).

### Image analysis

Two experienced nuclear medicine physicians read all of the images through consensus reading. They were blinded to the clinical and structural imaging findings. PET data were reconstructed using the ordered-subsets expectation maximization algorithm. The SUV was calculated according to the following formula: [measured activity concentration (Bq/mL) × body weight (g)] / injected activity (Bq). The frame on CT images of PET/CT was used to define the whole primary tumor. Regions of interest with a diameter of 1.5 cm were drawn on the pulmonary artery (for measurement of blood activity), heart, lung, liver, thyroid gland, spleen, bone, and muscle with the assistance of corresponding CT images. Because of the heterogeneity of tumor tissue, a maximum area of 3 × 3 pixels (7.04 × 7.04 mm) inside each tumor region was also determined, with an automated system to represent the highest radioactivity concentration in the tumor. The results were expressed in mean SUV. Finally, the tumor-to-blood (T/B) ratio, the ratio of the peak SUV of the tumor to the SUV of the pulmonary artery, was determined.

### Measurement of tumor size

All the patients measured the longest diameter of the primary tumor on CT images by two experienced nuclear medicine physicians. Patients were divided into 3 groups (< = 2cm; >2cm, < = 5cm; and >5cm) based on the longest diameter of tumor and further to investigate the correlation between different tumor size and hypoxia.

### Statistics

All data are presented as mean±SD unless otherwise stated. Statistical analysis was performed with the SPSS, version 17.0. The non-parametric Mann-Whitney U test was used to evaluate the significance of differences in the uptake of the markers between the control patients. Differences between continuous variables and dichotomous variables were tested by non-parametric Kruskal-Wallis H test. A *P* value of less than 0.05 was considered statistically significant.

## Results

### Patient characteristics and safety

The patient characteristics were summarized in **Table 1**. There was 1 patient missing the tumor diameter measurement because of obstructive pneumonia in the ^18^F-FETNIM study, and 1 patient with obstructive atelectasis in the ^18^F-FMISO study. There was no statistical difference in age, histopathology types, tumor size and staging between the two groups of patients, *P* = 0.452, 0.775, 0.471, 0.341.

**Table 1:** Demographic profile and histological diagnosis in 42 patients with lung cancer.

	^18^F-FETNIM PET/CT Patients(n)	^18^F-FMISO PET/CT Patients(n)	P
Total	18	24	
Histopathology			0.775
Adenocarcinoma	5	10	
Squamous carcinoma	10	8	
Large cell carcinoma	1	1	
Small cell lung cancer	2	5	
Tumor size			0.471
≤2cm	3	6	
>2cm, ≤5cm	9	12	
>5cm	5	5	
Staging			0.341
III	11	18	
IV	7	6	
Age, years(Median)	55.5(47–74)	59(38–76)	0.452

After the examination, there were no adverse or clinically detectable pharmacologic effects in any of the subjects. No significant changes in vital signs or the results of laboratory studies or electrocardiograms were observed.

### Biodistribution

The biodistribution of ^18^F-FETNIM and ^18^F-FMISO in the normal tissues is shown in **Table 2**. No significantly different uptake with ^18^F-FETNIM as compared to^18^F-FMISO was seen in muscle, thyroid, spleen, pancreas, heart, and lung. The highest accumulation activity was found in the kidneys, demonstrating renal clearance. The liver also showed moderate uptake. A significantly higher uptake with ^18^F-FETNIM as compared to^18^F-FETNIM was seen in blood, liver, and kidney, *P* = 0.007, <0.001, <0.001.

**Table 2:** Biodistribution of ^18^F-FETNIM and ^18^F-FMISO on PET/CT in Patients with Lung Cancer.

Tissue	^18^F-FETNIM(n = 18)	^18^F-FMISO(n = 24)	P
Muscle	1.43±0.26	1.30±0.24	0.140
Thyroid	1.31±0.16	1.40±0.27	0.263
Spleen	1.58±0.26	1.55±0.33	1.000
Pancreas	1.44±0.24	1.49±0.35	0.899
Heart	1.60±0.28	1.53±0.26	0.593
Lung	0.39±0.10	0.39±0.11	0.741
Blood	1.50±0.22	1.30±0.21	0.007
Liver	1.80±0.24	2.27±0.46	<0.001
Kidney	3.90±1.51	2.32±0.42	<0.001
Tumor	2.12±0.56	2.56±0.77	0.022
Tumor-to-Blood	1.42±0.33	1.98±0.54	<0.001

### Hypoxia detection for tumor

**Fig 1:**
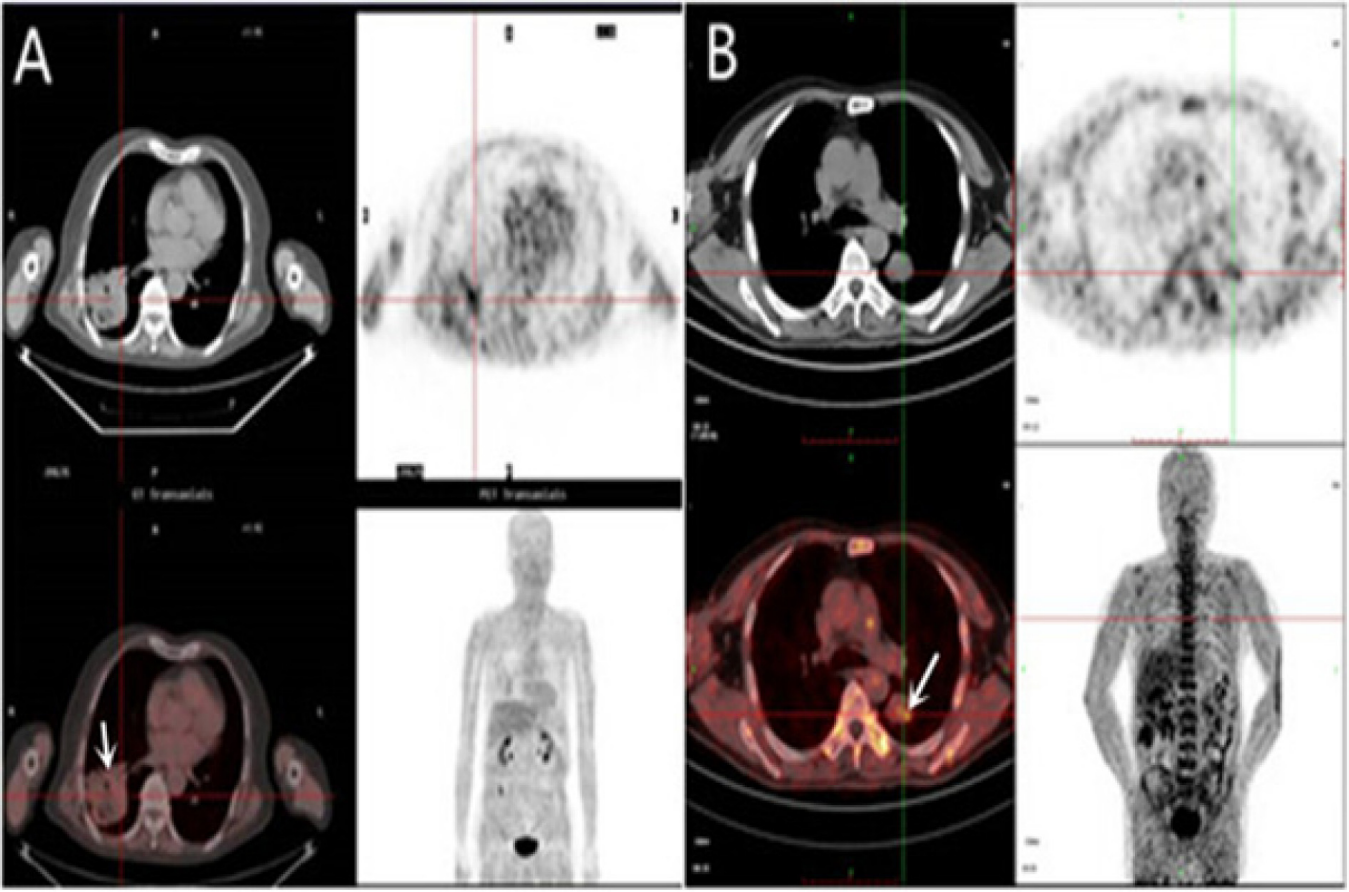
PET imaging. Major organs and regions uptake at 2 h after injection of ^18^F-FETNIM (**A**) in squamous carcinoma patient and ^18^F-FMISO (**B**) in small cell lung cancer patient. Arrows point to tumor.

The result of the SUV and T/B ratio measurement for tumors assessed by ^18^F-FETNIM and ^18^F-FMISO PET CT was shown in **Table 2** and **Fig 1**. Significantly higher SUV and T/B ratio with ^18^F-FMISO (2.56±0.77, 1.98±0.54) as compared to ^18^F-FETNIM (2.12±0.56, 1.42±0.33), were seen in tumor, *P =* 0.022, <0.001.

Semiquantitative assessment of ^18^F-FETNIM and ^18^F-FMISO uptake in tumor under different conditions was presented in **Table 3**. No significant difference of SUV (or T/B ratio) was observed between different histopathological subtypes both in ^18^F-FETNIM and ^18^F-FMISO in tumor, *P* = 0.121, 0.483 (or *P* = 0.221, 0.362). For ^18^F-FETNIM PET/CT, there was no correlation between the tumor size and the SUV (or T/B ratio) in tumors (*P* = 0.446, *P* = 0.707). With^18^F-FMISO, a significant increase in SUV (or T/B ratio) was seen along with the increase of tumor longest diameter (*P* = 0.015, *P* = 0.029). The SUV and T/B ratio were significantly higher in stage IV tumor than in stage III tumor for both ^18^F-FETNIM and ^18^F-FMISO, *P* = 0.021, 0.032, 0.013, 0.02.

**Table 3:** Semiquantitative Assessment of ^18^F-FETNIM and ^18^F-FMISO Uptake in Tumor Under Different Conditions.

Characteristic	SUV (mean±SD)		Tumor-to-Blood (mean±SD)	
	^18^F-FETNIM	^18^F-FMISO	^18^F-FETNIM	^18^F-FMISO
Histopathology				
Adenocarcinoma	2.63±0.72	2.95±0.64	1.61±0.54	2.13±0.48
Squamous carcinoma	1.90±0.28	2.21±0.67	1.32±0.15	1.69±0.59
Small Cell Lung Cancer	2.17±0.69	2.42±1.02	1.49±0.34	2.13±0.57
Large cell lung cancer	1.59	2.23	1.33	2.05
P value	0.121	0.221	0.483	0.362
Tumor size				
≤2cm	1.83±0.35	1.84±0.81	1.28±0.08	1.59±0.63
>2cm, ≤5cm	2.29±0.68	2.69±0.63	1.47±0.44	1.96±0.48
>5cm	1.96±0.42	3.19±0.41	1.39±0.20	2.49±0.10
P value	0.446	0.015	0.707	0.029
Staging				
III	1.88±0.34	2.41±0.81	1.27±0.21	1.87±0.57
IV	2.48±0.66	3.02±0.43	1.65±0.35	2.32±0.28
P value	0.021	0.032	0.013	0.02

## Discussion

42 patients with lung cancer were enrolled in the hypoxia imaging prospective study and our results indicated that ^18^F-FMISO PET/CT may play a better role in hypoxia detection in patients with lung cancer than that of ^18^F-FETNIM PET/CT. For clinical use of the hypoxia radiotracers, the PET signal needs to be quantified. Both for ^18^F-FMISO and ^18^F-FETNIM PET/CT considered T/B ratio threshold of a fixed value as a parameter to discriminate hypoxic from normoxic tissues [6,10].

In the biodistribution studies, there was no significantly different uptake between ^18^F-FETNIM and^18^F-FMISO on PET/CT in most of the normal tissues except for blood, kidneys, and liver. Hypoxia in tumors is a pathophysiologic consequence of structurally and functionally disturbed angiogenesis along with deterioration in the inability of oxygen to diffuse through tissues [11]. PET has the ability to differentiate individual tumor biology more accurately and modify treatment accordingly and the tracers targeting tissue hypoxia provides further tools to evaluate the molecular profile of tumors [12]. In this study, we validated the use of PET/CT in detection tumor hypoxia under different histopathological subtypes, stages, and tumor size with ^18^F-FMISO and ^18^F-FETNIM in patients with lung cancer.

In the current study, there was no difference between different histopathological subtypes with ^18^F-FMISO or ^18^F-FETNIM uptake in patients with lung cancer. For the ^18^F-FMISO study, the uptake in gliomas in correlation with histological findings was previously investigated by Cheret al [13]. Yamamoto et al had also found that there was a correlation between ^18^F-FMISO uptake and glioma grade, and T/B ratio in grade IV gliomas was significantly higher than all other grades [14]. Although ^18^F-FETNIM has been developed to evaluated hypoxia in human [15,16] tumors since 1995 [17], to our best knowledge, there are few reports about correlation between hypoxia and pathological subtypes in lung cancer.

^18^F-FMISO showed a potential advantage to discriminate the heterogeneous of oxygenation within the tumor and it also showed a significantly different T/B ratio or SUV than ^18^F-FETNIM in different tumor size in patients with lung cancer. The relationship between ^18^F-labelled hypoxia marker uptake and tumor size has been investigated in several preclinical studies [18,19]. Different from the current study, no significant correlation was reported between tumor size and uptake ratio in experimental glioma [18] or murine mammary carcinoma [19], when measured with ^18^F-FMISO. Cells become hypoxic when they exist at distances greater than 1–2 mm from vasculature, where diffusion of oxygen is no longer possible [20]. In theory, ^18^F-labelled hypoxia marker uptake will depend on size, vascularity, and developmental stage of the xenograph. Xenograft heterogeneity in mice model is smaller than that of patient, and that may explained why the tumor size has no correlation between tumor hypoxia. On the other hand, Chung et al [21] reported a moderate correlation between tumor weight and the tumor uptake of 18F-FETNIM in a murine sarcoma model. However, no different ^18^F-FETNIM uptake was observed between different tumor sizes in patient with lung cancer in this study.

We also analyzed the correlation between ^18^F-FMISO or ^18^F-FETNIM uptake and different stages in patient with inoperable stages III–IV lung cancer, and we found that T/B ratio or SUVmax in stage IV was significantly higher than stage III. As we all know, patient with stage IV lung cancer has a poor prognosis than stage III as hypoxia in tumors is associated with propagation and progression, as well as resistance to radiotherapy and some forms of chemotherapy [22–24]. Recent clinical studies have indicated that high ^18^F-FETNIM SUV may be predictive of treatment outcome in non–small cell lung cancer and esophageal squamous cell tumors [6,25]. The ability to determine the degree and extent of tumor hypoxia is important both as a prognostic biomarker [26] and as a means of selection of patients for hypoxia-directed therapies. The intratumor spatial distribution of hypoxia can potentially serve as a target in radiotherapy planning [27] and also indicate the use of a growing number of adjunct therapies [28]. In this study we further to validate the hypoxia detection ability of ^18^F-FMISO and ^18^F-FETNIM in patient with lung cancer.

Although this prospective study was performed successfully, it has the following deficiencies. The major drawback of the current study is that we have no definitive proof of the presence of hypoxia in the tumors of our series and larger cohorts are required to validate these findings in our study.

## Conclusion

In this study, we found that the uptake with^18^F-FMISO was significantly higher than ^18^F-FETNIM in tumors and might be a better hypoxia tracer for the patients with lung cancer.

## Supporting Information

S1 File Patient Characteristics and Imaging Data of ^18^F-FETNIM PET/CT. (DOCX)

S2 File Patient Characteristics and Imaging Data of ^18^F-FMISO PET/CT. (DOCX)
