# Supplementary material for: A Comparative Study of Noninvasive Hypoxia Imaging with 18F-Fluoroerythronitroimidazole and 18F-Fluoromisonidazole PET/CT in Patients with Lung Cancer
Source: PLoS One. 2016 Jun 20;11(6):e0157606. doi: 10.1371/journal.pone.0157606 (PMC4913930; doi:10.1371/journal.pone.0157606)
Supplement: S1 File — (DOCX) [file pone.0157606.s001.docx]

| **File 1** Patient Characteristics and Imaging Data of ^18^F-FETNIM PET/CT | | | | | | | | |
| --- | --- | --- | --- | --- | --- | --- | --- | --- |
| NO | Sex | Age | Tumor size | Staging | Histopathology | Tumor SUV | Blood SUV | Tumor/Blood |
| 1 | FM | 48 | 4.80 | IV | Adenocarcinoma | 3.71 | 1.68 | 2.21 |
| 2 | FM | 48 | 4.80 | IV | Adenocarcinoma | 2.94 | 1.44 | 2.05 |
| 3 | M | 64 | 7.30 | III | Small Cell Lung Carcinoma | 2.65 | 1.53 | 1.73 |
| 4 | M | 47 | 4.20 | IV | Squamous carcinoma | 2.18 | 1.48 | 1.47 |
| 5 | M | 47 | 4.20 | IV | Squamous carcinoma | 2.22 | 1.38 | 1.61 |
| 6 | M | 49 | unmeasurable | IV | Adenocarcinoma | 2.20 | 1.47 | 1.50 |
| 7 | M | 65 | 4.80 | IV | Adenocarcinoma | 2.43 | 1.66 | 1.46 |
| 8 | M | 53 | 5.50 | IV | Small Cell Lung Carcinoma | 1.68 | 1.34 | 1.25 |
| 9 | FM | 74 | 4.10 | III | Adenocarcinoma | 1.87 | 2.22 | 0.84 |
| 10 | M | 63 | 6.00 | III | Squamous carcinoma | 1.99 | 1.44 | 1.38 |
| 11 | M | 47 | 5.70 | III | Large cell lung carcinoma | 1.59 | 1.19 | 1.33 |
| 12 | M | 69 | 5.80 | III | Squamous carcinoma | 1.89 | 1.48 | 1.27 |
| 13 | M | 59 | 3.30 | III | Squamous carcinoma | 2.08 | 1.62 | 1.28 |
| 14 | M | 61 | 4.50 | III | Squamous carcinoma | 1.63 | 1.34 | 1.22 |
| 15 | F | 56 | 1.00 | III | Squamous carcinoma | 2.21 | 1.64 | 1.35 |
| 16 | M | 52 | 0.80 | III | Squamous carcinoma | 1.53 | 1.28 | 1.20 |
| 17 | M | 61 | 4.50 | III | Squamous carcinoma | 1.53 | 1.40 | 1.09 |
| 18 | M | 55 | 1.90 | III | Squamous carcinoma | 1.76 | 1.37 | 1.28 |
