# Supplementary material for: A Comparative Study of Noninvasive Hypoxia Imaging with 18F-Fluoroerythronitroimidazole and 18F-Fluoromisonidazole PET/CT in Patients with Lung Cancer
Source: PLoS One. 2016 Jun 20;11(6):e0157606. doi: 10.1371/journal.pone.0157606 (PMC4913930; doi:10.1371/journal.pone.0157606)
Supplement: S2 File — (DOCX) [file pone.0157606.s002.docx]

| **File 2** Patient Characteristics and Imaging Data of ^18^F-FMISO PET/CT | | | | | | | | |
| --- | --- | --- | --- | --- | --- | --- | --- | --- |
| NO | Sex | Age | Tumor size | Staging | Histopathology | Tumor SUV | Blood SUV | Tumor/Blood |
| 1 | M | 67 | 3.50 | III | Squamous carcinoma | 1.99 | 1.47 | 1.36 |
| 2 | M | 41 | 5.00 | IV | Adenocarcinoma | 3.23 | 1.30 | 2.47 |
| 3 | M | 41 | 5.60 | IV | Adenocarcinoma | 3.33 | 1.32 | 2.52 |
| 4 | M | 59 | 5.10 | IV | Adenocarcinoma | 3.39 | 1.39 | 2.45 |
| 5 | M | 59 | 4.00 | IV | Adenocarcinoma | 2.30 | 1.16 | 1.98 |
| 6 | M | 67 | 13.80 | III | Small Cell Lung Carcinoma | 3.64 | 1.38 | 2.64 |
| 7 | M | 54 | 3.97 | III | Adenocarcinoma | 1.98 | 1.61 | 1.23 |
| 8 | M | 54 | 3.97 | III | Adenocarcinoma | 2.38 | 1.60 | 1.49 |
| 9 | M | 38 | 1.00 | III | Small Cell Lung Carcinoma | 1.35 | 1.02 | 1.32 |
| 10 | M | 67 | 6.80 | III | Small Cell Lung Carcinoma | 2.54 | 1.07 | 2.37 |
| 11 | M | 72 | 5.90 | IV | Small Cell Lung Carcinoma | 3.14 | 1.24 | 2.54 |
| 12 | M | 52 | 1.00 | III | Small Cell Lung Carcinoma | 1.41 | 0.80 | 1.76 |
| 13 | FM | 59 | 4.80 | III | Squamous carcinoma | 2.38 | 1.44 | 1.65 |
| 14 | FM | 76 | 2.00 | III | Adenocarcinoma | 2.82 | 1.48 | 1.91 |
| 15 | M | 45 | 2.40 | III | Squamous carcinoma | 2.27 | 1.32 | 1.72 |
| 16 | M | 67 | 3.10 | III | Adenocarcinoma | 4.21 | 1.72 | 2.44 |
| 17 | M | 55 | unmeasurable | III | Large cell lung carcinoma | 2.23 | 1.09 | 2.05 |
| 18 | M | 75 | 3.00 | III | Adenocarcinoma | 2.98 | 1.12 | 2.67 |
| 19 | M | 62 | 2.90 | III | Adenocarcinoma | 2.85 | 1.30 | 2.18 |
| 20 | M | 55 | 1.90 | III | Squamous carcinoma | 1.68 | 1.15 | 1.46 |
| 21 | FM | 71 | 1.90 | III | Squamous carcinoma | 0.92 | 1.50 | 0.62 |
| 22 | M | 41 | 3.80 | IV | Squamous carcinoma | 2.70 | 1.40 | 1.94 |
| 23 | M | 59 | 3.20 | III | Squamous carcinoma | 2.90 | 1.26 | 2.31 |
| 24 | M | 54 | 2.00 | III | Squamous carcinoma | 2.87 | 1.15 | 2.49 |
